# Computation Methods for Biomedical Information Analysis

**DOI:** 10.1155/2018/8683601

**Published:** 2018-11-01

**Authors:** Yong Xia, Weidong Cai, Xiaofeng Yang, Shanshan Wang

**Affiliations:** ^1^School of Computer Science and Engineering, Northwestern Polytechnical University, Xi'an, China; ^2^School of Information Technologies, University of Sydney, Sydney, NSW 2006, Australia; ^3^Department of Radiation Oncology and Winship Cancer Institute, Emory University, Atlanta, GA 30322, USA; ^4^Shenzhen Institute of Advanced Technology, Chinese Academy of Sciences, Shenzhen 518055, China

With the advances in computer technology, biomedical data have been growing at an explosive speed, especially the massive accumulation of clinical medical data. It highly raises the requirement of data processing and analysis. Meanwhile, the computation methods can not only help medical professionals and researchers to get rid of the limitations of manual information processing in traditional methods but also provide them with more effective methods to use data. With the application of computation methods to biomedical information analysis, effective ways have been proposed to solve some problems that cannot be solved before.

This special issue provides a platform for medical professionals and researchers to present their ideas about the latest issues and developments in biomedical analysis fields. The papers in this special issue address the development and application of medical image segmentation, classification, detection, modeling, predicting, and literature mining.

The major part of this special issue is a collection of papers on image processing and analysis in various application areas.

The paper “Predicting Interactions between Virus and Host Proteins Using Repeat Patterns and Composition of Amino Acids” uses the repeat patterns and composition of amino acids to predict interactions between virus and host proteins and find new virus-host protein-protein interactions (PPIs) with little information.

The paper “Bayesian Classification Models for Premature Ventricular Contraction Detection on ECG Traces” compares three well-known Bayesian classification algorithms, including the naive Bayes, linear discriminant analysis, and quadratic discriminant analysis algorithms, in distinguishing the normal heartbeats, premature ventricular contraction beats, and others.

The paper “Organic Boundary Location Based on Color-Texture of Visual Perception in Wireless Capsule Endoscopy Video” combines the color-saliency region detection method and support vector machine (SVM) classifier to promote the efficiency and accuracy of locating the pylorus in wireless capsule endoscopy videos.

The paper “Breast Mass Detection in Digital Mammogram Based on Gestalt Psychology” incorporates visual perception properties described by the Gestalt psychology framework into breast mass detection in a digital mammogram to achieve good performance on both the Mammographic Image Analysis Society (MIAS) data set and Digital Database for Screening Mammography (DDSM) data set.

The paper “A Filtering Method for Identification of Significant Target mRNAs of Coexpressed and Differentially Expressed MicroRNA Clusters” devises to use hypergeometric distributions to identify significant miRNA target genes from an extensive list of predicted miRNA target gene relationships.

The paper “A Method for Tooth Model Reconstruction Based on Integration of Multimodal Images” develops a tooth model reconstruction method based on integration of computed tomography (CT) images and laser scan images, which can generate a tooth model with a more accurate crown and can obtain a complete tooth model at any stage of orthodontic treatment by using one CT scan at the pretreatment stage and one laser scan at that stage to avoid multiple CT scans.

The paper “Link Prediction Investigation of Dynamic Information Flow in Epilepsy” adapts link prediction for proposing a novel workflow, which detects the seizure occurrence and monitors the total seizure course for quantifying seizure dynamics and uncovers pathological mechanisms of epilepsy.

The paper “Comparative Study on Automated Cell Nuclei Segmentation Methods for Cytology Pleural Effusion Images” presents a comparative study on 12 automated cell nuclei segmentation methods, finding that the segmentation performances of the Otsu, k-means, mean shift, Chan–Vese, and graph cut methods outperform the others, which will be useful for current and potential future studies on cytology images of pleural effusion.

This special issue also has two papers, which describe medical data retrieval systems.

The paper “The Cell Research Trends of Asthma: A Stem Frequency Analysis of the Literature” summarizes the asthma literature indexed in the medical literature analysis and retrieval system online (MEDLINE) and explores the history to present trends of asthma cell research by stem frequency ranking to forecast the prospect of the future work.

The paper “The Wall Apposition Evaluation for a Mechanical Embolus Retrieval Device” presents a computational evaluation approach to the wall apposition of a cerebral mechanical emboli retrieval device (MERD) and provides references and suggestions for further research and work.

Although it is impossible to comprehensively cover the growing field of computation methods for biomedical information analysis in a special issue like this, we believe that this special issue presents a set of state-of-the-art computational methods and their potentials in the biomedical information analysis and related domains. We hope this issue would inspire further research on the aforementioned topics.

